# Psychotropic medication use pre and post-diagnosis of cluster B personality disorder: a Quebec’s health services register cohort

**DOI:** 10.3389/fpsyt.2023.1243511

**Published:** 2023-11-23

**Authors:** Carlotta Lunghi, Lionel Cailhol, Victoria Massamba, Elhadji A. Laouan Sidi, Caroline Sirois, Elham Rahme, Louis Rochette, Suzane Renaud, Evens Villeneuve, Marion Koch, Robert Biskin, Cathy Martineau, Philippe Vincent, Pierre David, Alain Lesage

**Affiliations:** ^1^Department of Health Sciences, Université du Québec à Rimouski, Lévis, QC, Canada; ^2^Department of Medical and Surgical Sciences, University of Bologna, Bologna, Italy; ^3^Institut National de Santé Publique du Québec, Quebec, QC, Canada; ^4^Department of Psychiatry and Research Center, Institut Universitaire de Santé Mentale de Montréal, Montreal, QC, Canada; ^5^Department of Psychiatry and Addiction, Université de Montréal, Montreal, QC, Canada; ^6^Faculty of Pharmacy, Université Laval, Quebec, QC, Canada; ^7^Division of Clinical Epidemiology, Department of Medicine, McGill University, Montreal, QC, Canada; ^8^CISSS des Laurentides et CISSS des Iles, Saint-Jérôme, QC, Canada; ^9^Faculty of Medicine, Université Laval, Quebec, QC, Canada; ^10^Institut universitaire en santé mentale de Québec, Quebec, QC, Canada; ^11^Department of Psychiatry, McGill University, Montreal, QC, Canada; ^12^Department of Psychiatry, Hôpital de Gatineau, Gatineau, QC, Canada; ^13^Faculty of Pharmacy, Université de Montréal, Montreal, QC, Canada

**Keywords:** cluster B personality disorders, borderline personality disorder, histrionic personality disorder, narcissistic personality disorder, antisocial personality disorder, pharmacotherapy, drug utilization, psychiatric medications

## Abstract

**Background:**

Cluster B personality disorders (PDs) are considered some of the most severe mental health conditions. Scarce evidence exists about the real-world utilization of psychotropics for cluster B PD individuals.

**Objective:**

We aimed to uncover trends and patterns of psychotropic medication use among individuals diagnosed with cluster B PD in the year before and after their diagnosis and to identify factors associated with medication use in a large cohort of individuals newly diagnosed with cluster B PDs.

**Methods:**

We conducted a population-based observational study using Quebec’s health services register. We identified Quebec residents aged ≥14 years and insured with the provincial drug plan with a first diagnosis of cluster B PD recorded between April 1, 2002, and March 31, 2019. Cluster B PD was defined with ICD-9/10 diagnostic codes. We retrieved all claims for the main psychotropic medication classes: antipsychotics, antidepressants, anxiolytics, mood stabilizers, and attention-deficit/hyperactivity disorder (ADHD) medications. We calculated the proportion of individuals exposed to these medication classes and analyzed trends over the years using robust Poisson regression models, adjusting for potential confounders. We used robust Poisson regression to identify factors associated with medication class use.

**Results:**

We identified 87,778 new cases of cluster B PD, with a mean age of 44.5 years; 57.5% were women. Most frequent psychiatric comorbidities in the five years before cluster B PD diagnosis were depression (50.9%), anxiety (49.7%), and psychotic disorders (37.5%). Most individuals (71.0%) received at least one psychotropic during the year before cluster B PD diagnosis, and 78.5% received at least one of these medications in the subsequent year. The proportion of users increased after the diagnosis for antidepressants (51.6–54.7%), antipsychotics (35.9–45.2%), mood stabilizers (14.8–17.0%), and ADHD medications (5.1–5.9%), and remained relatively stable for anxiolytics (41.4–41.7%). Trends over time showed statistically significant increased use of antipsychotics and ADHD medications, decreased use of anxiolytics and mood stabilizers, and a stable use of antidepressants.

**Conclusion:**

Psychotropic medication use is highly prevalent among cluster B PD individuals. We observed an increase in medication use in the months following the diagnosis, particularly for antipsychotics, antidepressants, and mood stabilizers.

## 1 Introduction

Cluster B personality disorders (PDs) are severe and chronic mental health conditions characterized by relational and affective instability, identity disorder, and marked impulsivity (1). People with cluster B PDs present several difficulties in their relational and occupational functioning, decreasing their quality of life (1). A recent systematic review and meta-analysis estimated the lifetime prevalence of cluster B PDs at 5.5%, with differences between subtypes (0.8% for histrionic, 1.2% for narcissistic, 1.9% for borderline, and 3.1% for antisocial) (2). In Quebec, Canada, we estimated cluster B PDs lifetime prevalence at 2.6% (3). Individuals with cluster B PDs are high users of medical services, more than schizophrenia patients (4). They also have high rates of comorbid substance use disorders, particularly alcohol, opioid, and cocaine use disorders (5). Additionally, they exhibit a reduced life expectancy, and 20.5% of premature mortality is attributable to suicide (3, 6, 7). Indeed, borderline personality disorder may specifically indicate a predisposition to suicidal behavior disorder diagnoses (8).

Treatment of cluster B PDs is a clinical challenge because of the high suicidal behaviors and the resistance of clinical symptoms to pharmacological treatments (9, 10). Current clinical guidelines, such as the 2009 United Kingdom’s National Institute for Health Care and Excellence (NICE) recommendations (11) or the European guidelines for PDs (12), and a recent systematic review of clinical recommendations on the treatment of PD patients made by different mental health organizations worldwide (13) do not recommend the use of pharmacotherapy for the treatment of cluster B PD individuals because of the lack of evidence to support their use. Systematic reviews have been conducted in the last decade to identify which medications could benefit patients with cluster B PDs (10, 14–16). A 2010 Cochrane review of randomized clinical trials (RCTs) assessing the effectiveness and safety of different psychotropic medication classes found only fragmentary evidence supporting the use of antipsychotics (especially first generation) in reducing anger (14). More recent reviews (10, 16) and Cochrane updates (17, 18) did not find substantial differences from newer RCTs evaluating second-generation antipsychotics, antidepressants, mood stabilizers, or various medications (e.g., the antiepileptic lamotrigine or the anti-dementia drug memantine). Indeed, no medication has been officially approved for treating patients with cluster B PDs.

Although systematic reviews (10, 18, 19), expert opinions (9), and clinical practice guidelines (11, 12) recognize psychotherapy as the first-line treatment for cluster B PDs, there is evidence of a gap between evidence-based recommendations on pharmacotherapy and current clinical practice, with different psychotropic medications prescribed to treat this condition despite the lack of evidence for their efficacy. Indeed, some studies have reported high consumption of psychotropics in individuals with cluster B PDs (20–31). Nevertheless, most of these studies were cross-sectional (21, 22, 26, 28, 30), focused on borderline individuals (21–23, 25, 26, 28, 31), were conducted in small samples (26, 31), or considered only hospitalized patients (25, 26, 31), those participating in mental health programs (20, 22, 23, 29), or those followed by a psychiatrist (21).

The aim of this study was thus to draw a portrait of the use of psychotropic medications in individuals with cluster B PDs from a publicly managed care system enrolling all 8.5 million inhabitants of the Canadian province of Quebec. Specific objectives were (1) to describe psychotropic medication use in the year before and after cluster B PD diagnosis; (2) to identify trends and patterns in psychotropic use over 16 years; and (3) to identify factors associated with the exposure to psychotropic medication classes.

## 2 Materials and methods

### 2.1 Study design and data source

We conducted a population-based cohort study of Quebec residents covered under the universal provincial health program (32) using medico-administrative data from the Quebec Integrated Chronic Disease Surveillance System (QICDSS) database (33). More than 99% of the Quebec population is covered under this provincial health program and is included in the QICDSS database. QICDSS comprises claims data from physician visits and hospitalizations starting from January 1, 1996. Diagnoses from physician visits and hospitalizations are based on the ninth and tenth revisions of the International Classification of Diseases (ICD-9 and ICD-10). QICDSS also comprises the death registry. Moreover, this database contains information on reimbursed medications for all residents registered under the public drug insurance plan, namely those without a private drug insurance plan, those on a guaranteed income supplement or welfare, and all citizens aged 65 years and older not living in long-term care facilities. In 2021, the public drug plan covered about 3.75 million people (43.5% of the Quebec population) (32).

### 2.2 Population

We identified all Quebec residents ages 14 years old and older with a first cluster B PD diagnosis recorded in the QICDSS database between April 1, 2002, and March 31, 2019. To identify cluster B PD patients in the QICDSS database, we used a case definition that was previously developed by a team of experts (four psychiatrists and one psychologist experienced in treating PDs in Quebec) and described elsewhere (3). Briefly, following two multisite work sessions, the team of experts was able to achieve a consensus on the definitive list of ICD-9 and ICD-10 codes typically employed in the diagnoses of histrionic, narcissistic, borderline, or antisocial personality disorders in Quebec. The cluster B PD case was defined as any patient with at least one ICD-9 (301.1, 301.3, 301.5, 301.7, 301.8, or 301.9) or ICD-10 (F070, F340, F341, F488, F602, F603, F604, F606, F608, F609, F61, F620, F621, F628, F629, F681, F688, F69) diagnostic code. The choice of the ICD codes was based on the practice experience of the expert team to obtain the most clinically relevant data. Indeed, all selected ICD-9 and ICD-10 codes were meant to identify core symptoms of cluster B PDs symptoms based on the DSM. The expert panel also decided on the inclusion of the ICD-9 code 301.9 (unspecified PD) among the codes considered for the case definition, deeming it commonly used for borderline PD in Quebec. To collect information on prescribed medications, we excluded all the cluster B PD individuals who were not fully covered under the public drug plan starting from 1 year before to 1 year after the PD diagnosis. Information on prescribed drugs is unavailable in the QICDSS database for individuals with private medical insurance.

### 2.3 Medication exposure

We included all medications listed in the Quebec public drug plan during the study period to identify psychotropic medications claimed during the period going from one year before to one year after cluster B PD diagnosis. We further classified drugs into five main psychotropic medication classes: antipsychotics, antidepressants, anxiolytics, mood stabilizers, and attention-deficit/hyperactivity disorder (ADHD) medications, according to the American Hospital Formulary Service (AHFS) classification (34) and common drug denomination (chemical name of the medication), as reported in Supplementary Table S1. We considered that an individual was exposed to a medication class if they claimed at least one medication from this class in the one year before or after cluster B PD diagnosis.

### 2.4 Sociodemographic variables

Baseline socio-demographic variables, assessed at the diagnosis date, included sex, age group, material and social deprivation indexes (in quintiles), and the geographical area based on the Quebec census (Montreal census metropolitan area (CMA), >1,000,000 inhabitants; Other CMA, 100,000-1,000,000 inhabitants; Agglomerations, 10,000–100,000 inhabitants; and Small town/rural area, <10,000 inhabitants). Material and social deprivation indexes are ecological indexes based on the census dissemination area divided into quintiles and represent a proxy of the individual’s socioeconomic status (35). The first quintile includes the least deprived, and the fifth quintile the most disadvantaged areas. For psychiatric conditions, an individual was considered to have a comorbid disorder if they had a claim for a physician visit or a hospitalization with an ICD-9 or ICD-10 code related to that condition recorded in the QICDSS in the five years before cluster B PD diagnosis.

### 2.5 Statistical analyses

We used descriptive statistics to document the baseline characteristics of the study cohort (age, sex, social and material deprivation, year of diagnosis, and geographical area) and clinical information (psychiatric diagnosis and the number of psychiatric comorbidities) according to age groups (14–24 years; 25–49 years; 50–64 years; 65 years and above). Demographics were reported as mean and standard deviation for age and proportions and 99% confidence intervals (CIs) for categorical variables. For each subject, we assessed the number of different psychotropic medications claimed in the year before and the year after the diagnosis of cluster B PD by using the common drug denominations (identifying the chemical entity). We calculated the proportion of individuals exposed to the main psychotropic medication classes (antipsychotics, antidepressants, anxiolytics, mood stabilizers, and ADHD medications).

We tested trends of change in the prevalence of psychotropic medication class use in the year after cluster B PD diagnosis according to the year of diagnosis using robust Poisson regression models, adjusting for age, sex, material and social deprivation, geographical area, and psychiatric comorbidities. Using robust Poisson regression analyses, we further identified the clinical and socio-demographic factors associated with the use of medication classes in the year after cluster B PD diagnosis, modeling the number of individuals who claimed at least one medication in the psychotropic class under study in that specific model. We calculated unadjusted and adjusted prevalence ratios (PRs) with their 99% CIs. We performed all the analyses using SAS Enterprise Guide 7.1.

## 3 Results

We identified 211,974 individuals aged 14 years and above with a first diagnosis of cluster B PD between the fiscal years 2002–2003 and 2018–2019. Among them, we excluded those not fully covered by the public drug plan between 1 year before and one year after the diagnosis. Thus, we gathered a group of 87,778 people, with 6.7% of them diagnosed during a hospital stay, while the remaining 93.3% diagnosed during an outpatient visit (45.7% by a general practitioner and 44.3% by a psychiatrist) and classified according to the fiscal year of their first diagnosis. The general practitioners are commonly available in the Emergency Rooms of hospitals where PD are more likely to visit than patients with other diseases, such as schizophrenia (4). The cohort was predominantly female and socially and materially deprived. Characteristics of the study population are reported in Table 1 according to the individual’s age group at the time of cluster B PD diagnosis.

**Table 1:** Sociodemographic and clinical characteristics of the cohort of individuals at the time of a first diagnosis of cluster B personality disorder.

Characteristics	14–24 years (*N* = 16,317)			25–49 years (*N* = 37,825)			50–64 years (*N* = 17,346)			65+ years (*N* = 16,290)		
	*N* ^#^	%	(99% CI)	N^#^	%	(99% CI)	N^#^	%	(99% CI)	N^#^	%	(99% CI)
Age in years – Mean (SD)	19.7	(3.1)	-	36.6	(7.4)	-	56.4	(4.3)	-	75.0	(7.4)	-
Sex												
Female	9,850	60.4	(59.4–61.4)	20,710	54.8	(54.1–55.4)	9,795	56.5	(55.5–57.4)	10,070	61.8	(60.8–62.8)
Male	6,465	39.6	(38.6–40.6)	17,110	45.2	(44.6–45.9)	7,550	43.5	(42.6–44.5)	6,215	38.2	(37.2–39.2)
Material deprivation^*^ (quintile)												
1 (least deprived)	1885	11.6	(10.9–12.2)	4,505	11.9	(11.5–12.3)	1950	11.2	(10.6–11.9)	2,465	15.1	(14.4–15.9)
2	2,435	14.9	(14.2–15.6)	5,500	14.5	(14.1–15.0)	2,520	14.5	(13.8–15.2)	2,615	16.0	(15.3–16.8)
3	3,110	19.1	(18.3–19.9)	7,085	18.7	(18.2–19.3)	3,140	18.1	(17.4–18.9)	2,790	17.1	(16.4–17.9)
4	3,600	22.1	(21.2–22.9)	8,645	22.9	(22.3–23.4)	3,775	21.8	(21.0–22.6)	3,130	19.2	(18.4–20.0)
5 (most deprived)	4,535	27.8	(26.9–28.7)	10,135	26.8	(26.2–27.4)	4,725	27.2	(26.4–28.1)	3,180	19.5	(18.7–20.3)
Missing	750	4.6	(4.2–5.0)	1955	5.2	(4.9–5.5)	1,240	7.1	(6.6–7.6)	2,110	12.9	(12.3–13.6)
Social deprivation^*^ (quintile)												
1 (least deprived)	2065	12.7	(12.0–13.3)	3,955	10.4	(10.0–10.9)	1780	10.3	(9.7–10.9)	1875	11.5	(10.9–12.2)
2	2,375	14.6	(13.9–15.3)	5,005	13.2	(12.8–13.7)	2,385	13.7	(13.1–14.4)	2,265	13.9	(13.2–14.6)
3	2,755	16.9	(16.1–17.6)	5,975	15.8	(15.3–16.3)	2,765	15.9	(15.2–16.6)	2,620	16.1	(15.3–16.8)
4	3,455	21.2	(20.4–22.0)	8,425	22.3	(21.7–22.8)	3,520	20.3	(19.5–21.1)	3,240	19.9	(19.1–20.7)
5 (most deprived)	4,910	30.1	(29.2–31.0)	12,515	33.1	(32.5–33.7)	5,665	32.6	(31.7–33.6)	4,175	25.6	(24.7–26.5)
Missing	750	4.6	(4.2–5.0)	1950	5.2	(4.9–5.5)	1,240	7.1	(6.6–7.6)	2,110	12.9	(12.3–13.6)
Geographical area^$^												
Montreal CMA	7,570	46.4	(45.4–47.4)	17,020	45.0	(44.3–45.7)	7,160	41.3	(40.3–42.2)	6,720	41.2	(40.3–42.2)
Other CMA	3,175	19.5	(18.7–20.3)	7,880	20.8	(20.3–21.4)	3,680	21.2	(20.4–22.0)	3,695	22.7	(21.8–23.5)
Agglomeration	2,385	14.6	(13.9–15.3)	5,265	13.9	(13.5–14.4)	2,460	14.2	(13.5–14.9)	2,435	14.9	(14.2–15.7)
Small town/rural area	3,160	19.4	(18.6–20.2)	7,475	19.8	(19.2–20.3)	3,810	22.0	(21.1–22.8)	3,335	20.5	(19.7–21.3)
Missing	25	0.2	(0.1–0.2)	185	0.5	(0.4–0.6)	235	1.3	(1.1–1.6)	105	0.6	(0.5–0.8)
Psychotropic medications^+^												
Antipsychotics	5,350	32.8	(31.8–33.7)	14,170	37.5	(36.8–38.1)	6,785	39.1	(38.2–40.1)	5,255	32.3	(31.3–33.2)
Antidepressants	6,235	38.2	(37.2–39.2)	20,155	53.3	(52.6–54.0)	10,350	59.7	(58.7–60.6)	8,545	52.4	(51.4–53.5)
Anxiolytics	2,905	17.8	(17.0–18.6)	14,895	39.4	(38.7–40.0)	9,545	55.0	(54.0–56.0)	9,020	55.4	(54.4–56.4)
Mood stabilizers	1,380	8.4	(7.9–9.0)	5,695	15.1	(14.6–15.5)	3,615	20.8	(20.1–21.6)	2,335	14.3	(13.6–15.0)
ADHD medications	2,490	15.3	(14.5–16.0)	1,590	4.2	(3.9–4.5)	305	1.8	(1.5–2.0)	95	0.6	(0.4–0.7)
Psychiatric comorbidity^&^												
Schizophrenia	1,235	7.6	(7.0–8.1)	4,470	11.8	(11.4–12.2)	2,370	13.6	(13.0–14.3)	1,095	6.7	(6.2–7.2)
Other psychosis	1715	10.5	(9.9–11.1)	4,540	12.0	(11.6–12.4)	2,135	12.3	(11.7–13.0)	2080	12.8	(12.1–13.4)
Depression	6,950	42.6	(41.6–43.6)	21,510	56.9	(56.2–57.5)	9,655	55.7	(54.7–56.6)	6,520	40.0	(39.0–41.0)
Bipolar disorders	2,240	13.7	(13.0–14.4)	8,450	22.3	(21.8–22.9)	4,375	25.2	(24.4–26.1)	2,790	17.1	(16.4–17.9)
Anxiety disorders	6,465	39.6	(38.6–40.6)	20,460	54.1	(53.4–54.7)	9,135	52.7	(51.7–53.6)	7,575	46.5	(45.5–47.5)
Adaptive disorders	4,310	26.4	(25.5–27.3)	10,890	28.8	(28.2–29.4)	4,470	25.8	(24.9–26.6)	2,860	17.6	(16.8–18.3)
Alcohol abuse disorders	1,630	10.0	(9.4–10.6)	6,135	16.2	(15.7–16.7)	2,865	16.5	(15.8–17.3)	1705	10.5	(9.9–11.1)
Drug abuse disorders	2,920	17.9	(17.1–18.7)	8,700	23.0	(22.4–23.6)	2,445	14.1	(13.4–14.8)	900	5.5	(5.0–6.0)
ADHD	2,230	13.7	(13.0–14.3)	1,030	2.7	(2.5–2.9)	150	0.9	(0.7–1.0)	60	0.4	(0.2–0.5)
Eating disorders	160	1.0	(0.8–1.2)	95	0.3	(0.2–0.3)	20	0.1	(0.0–0.2)	15	0.1	(0.0–0.2)
Number of psychiatric comorbidities^&^												
0	3,910	24.0	(23.1–24.8)	5,525	14.6	(14.1–15.1)	2,740	15.8	(15.1–16.5)	4,605	28.3	(27.3–29.2)
1–2	7,550	46.3	(45.3–47.3)	16,780	44.4	(43.7–45.0)	7,875	45.4	(44.4–46.4)	7,715	47.4	(46.3–48.4)
3–4	3,700	22.7	(21.8–23.5)	11,710	31.0	(30.3–31.6)	5,270	30.4	(29.5–31.3)	3,355	20.6	(19.8–21.4)
5–6	975	6.0	(5.5–6.5)	3,360	8.9	(8.5–9.3)	1,330	7.7	(7.2–8.2)	580	3.6	(3.2–3.9)
7+	180	1.1	(0.9–1.3)	445	1.2	(1.0–1.3)	130	0.7	(0.6–0.9)	40	0.2	(0.1–0.3)
Fiscal year^+^ of B-PD diagnosis												
2002	790	4.8	(4.4–5.3)	2,660	7.0	(6.7–7.4)	1,050	6.1	(5.6–6.5)	865	5.3	(4.9–5.8)
2003	780	4.8	(4.4–5.2)	2,565	6.8	(6.4–7.1)	1,010	5.8	(5.4–6.3)	780	4.8	(4.4–5.2)
2004	810	4.9	(4.5–5.4)	2,445	6.5	(6.1–6.8)	1,035	6.0	(5.5–6.4)	765	4.7	(4.3–5.1)
2005	770	4.7	(4.3–5.1)	2,460	6.5	(6.2–6.8)	955	5.5	(5.1–6.0)	780	4.8	(4.4–5.2)
2006	800	4.9	(4.5–5.3)	2,295	6.1	(5.8–6.4)	965	5.6	(5.1–6.0)	830	5.1	(4.7–5.6)
2007	795	4.9	(4.4–5.3)	2,375	6.3	(6.0–6.6)	1,080	6.2	(5.8–6.7)	925	5.7	(5.2–6.1)
2008	820	5.0	(4.6–5.5)	2,305	6.1	(5.8–6.4)	1,050	6.1	(5.6–6.5)	935	5.7	(5.3–6.2)
2009	850	5.2	(4.8–5.7)	2,295	6.1	(5.7–6.4)	995	5.8	(5.3–6.2)	925	5.7	(5.2–6.1)
2010	885	5.4	(5.0–5.9)	2,240	5.9	(5.6–6.2)	1,025	5.9	(5.4–6.4)	950	5.8	(5.4–6.3)
2011	970	6.0	(5.5–6.4)	2,325	6.2	(5.8–6.5)	1,175	6.8	(6.3–7.3)	1,050	6.4	(5.9–6.9)
2012	1,170	7.2	(6.7–7.7)	2,250	6.0	(5.6–6.3)	1,170	6.7	(6.2–7.2)	1,085	6.7	(6.2–7.2)
2013	1,285	7.9	(7.3–8.4)	2,245	5.9	(5.6–6.3)	1,130	6.5	(6.1–7.0)	1,180	7.2	(6.7–7.8)
2014	1,320	8.1	(7.5–8.6)	2,300	6.1	(5.8–6.4)	1,110	6.4	(5.9–6.9)	1,120	6.9	(6.4–7.4)
2015	1,235	7.6	(7.1–8.1)	2,145	5.7	(5.4–6.0)	1,090	6.3	(5.8–6.8)	1,110	6.8	(6.3–7.3)
2016	1,140	7.0	(6.5–7.5)	1970	5.2	(4.9–5.5)	1,075	6.2	(5.7–6.7)	1,240	7.6	(7.1–8.1)
2017	910	5.6	(5.1–6.0)	1,465	3.9	(3.6–4.1)	760	4.4	(4.0–4.8)	930	5.7	(5.3–6.2)
2018	985	6.0	(5.6–6.5)	1,485	3.9	(3.7–4.2)	655	3.8	(3.4–4.1)	820	5.0	(4.6–5.5)

ADHD, attention-deficit/hyperactivity disorder; CI, confidence interval; CMA, census metropolitan area; B-PD, cluster B personality disorder. ^#^Randomly rounded to 0/5; B-PD ^*^Pampalon index at cluster B personality disorder (PD) diagnosis; ^$^Montreal CMA: Montreal and Laval; Other CMA: Quebec, Sherbrooke, Trois-Rivières, Saguenay and Gatineau; ^+^Psychotropic medications claimed in the year before B-PD diagnosis; ^&^Psychiatric comorbidities in the five years before B-PD diagnosis; ^+^Fiscal years are comprised between the 1st of April of one year and the 31st of March of the following year.

### 3.1 Medication exposure before and after cluster B PD diagnosis

The proportion of individuals exposed to any psychotropic medication during the year before and after the cluster B PD diagnosis was generally high. It varied only slightly during the study period, between 72.0 and 75.5% before and 77.0 and 80.2% after the diagnosis, depending on the year. These proportions were also constantly higher compared to the proportion of individuals exposed to the same medication classes the year before the diagnosis. The mean number of different psychotropics used has decreased slightly over the study period, from 3.1 in 2002 to 2.8 in 2018 (Supplementary Figure S1). The mean number of antidepressants and mood stabilizers remained relatively stable from 2002 to 2018 (1.53 to 1.51 and 1.22 to 1.18, respectively), while that of antipsychotics and anxiolytics decreased (1.45 to 1.39 and 1.47 to 1.22, respectively), and that of ADHD medications increased (1.05 to 1.19) during the same period.

**Figure 1:**
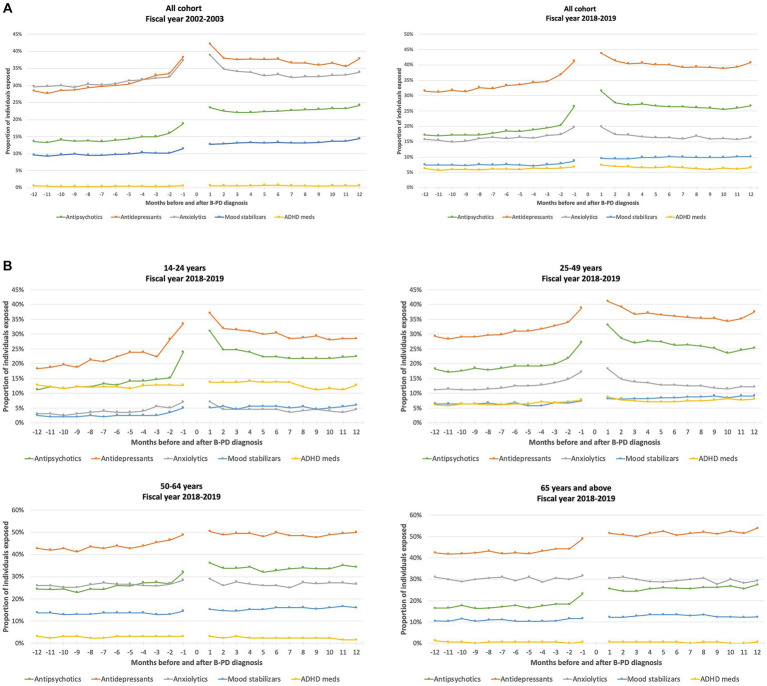
Proportion of individuals exposed to different classes of psychotropic medications in the 12 months before and after a cluster B personality disorder (B-PD) diagnosis, by month (fiscal years 2002–2003 and 2018–2019) **(A)**, and according to the age group, by month (fiscal year 2018–2019) **(B)**.

Figure 1 (panel A) reports the monthly proportion of individuals in the cohort exposed to different psychotropic medication classes during the year before and year after the diagnosis of cluster B PD. We could identify an increase in antidepressant and antipsychotic prescriptions nearby the cluster B PD diagnosis, which was substantially maintained in subsequent months. The use of anxiolytics slightly increased around PD diagnosis, but this increase returned to the previous level within a few months. On the contrary, the other classes remained stable before and after cluster B PD diagnosis. Figure 1 also shows the monthly proportion of individuals exposed to different psychotropic medication classes according to age groups. Antidepressants were the most frequently used medications in all age groups, while ADHD medications were mainly used in the youngest group and anxiolytics in the oldest one. An increase in use nearby the time of cluster B PD diagnosis was evident, especially for antidepressants and antipsychotics in all age groups, but particularly marked in younger individuals.

### 3.2 Trends and patterns in medication exposure during the 16-year study period

**Figure 2:**
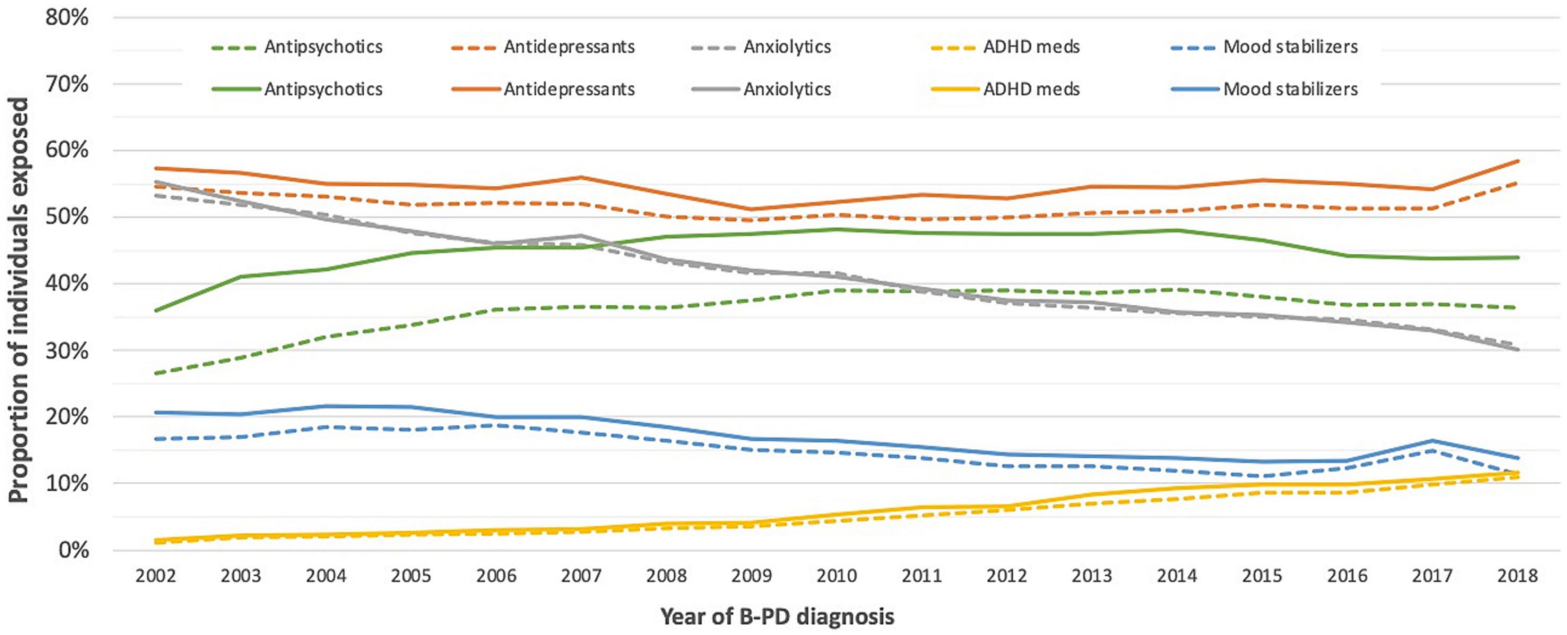
Proportion of individuals exposed to different classes of psychotropic medications in the year before and after a cluster B personality disorder diagnosis.

Figure 2 reports the proportion of individuals exposed to the different psychotropic medication classes according to the cluster B PD diagnosis year. Over the study period, the exposure to antidepressants increased slightly, while the increase in exposure to antipsychotics and ADHD medications was more pronounced. Mood stabilizers and, notably, anxiolytics decreased. As for antipsychotics, the growth was driven by atypical antipsychotics (Supplementary Figure S2).

Sex- and age-adjusted trends over time showed a statistically significant rise in the use of antidepressants (yearly mean change of individuals exposed: +0.51%; 99% CI: +0.36%, +0.67%; *p* value < 0.0001), antipsychotics (+1.61%; 99% CI: +1.43%, +1.79%; *p* value < 0.0001), and ADHD medications (+13.1%; 99% CI: +12.5%, +13.8%; *p* value < 0.0001). On the contrary, anxiolytics showed a sharp decrease (−3.795%; 99% CI: −3.97%, −3.61%; *p* value < 0.0001), and mood stabilizers a less pronounced one (−2.47%; 99% CI: −2.8%, −2.14%; *p* value < 0.0001).

**Figure 3:**
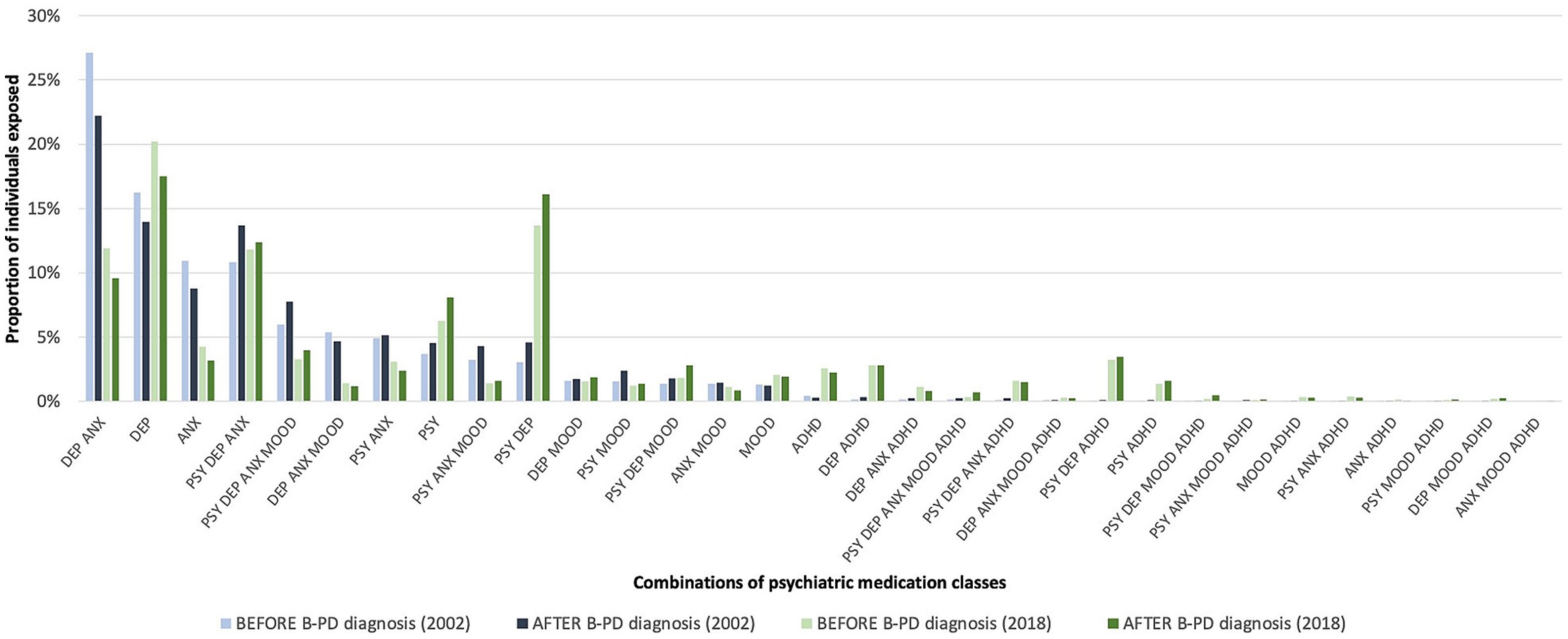
Proportion of individuals exposed to combinations of psychotropic medication classes in the year before and after a cluster B personality disorder (B-PD) diagnosis (2002 vs. 2018).

Quite a significant proportion of individuals were exposed to combinations of psychotropic medication classes (Figure 3; Supplementary Table S1). The more frequent combination in 2002 (with more than 20% of individuals exposed) was antidepressants and anxiolytics. In 2018, this combination was used by less than 10% of individuals. In recent years, combinations containing ADHD medications have become more frequent. Another more-used combination in 2018 than 2002 was antipsychotics with antidepressants, reaching 15% of usage in 2018.

### 3.3 Factors associated with exposure to psychotropic medications

When analyzing factors associated with antipsychotics, antidepressants, anxiolytics, mood stabilizers, or ADHD medications exposure in the year after cluster B PD diagnosis, we found statistically significant differences according to the medication class for age, sex, social and material deprivation, area of residence and psychiatric comorbidities (Table 2).

**Table 2:** Multivariate robust Poisson regression analyses of factors associated with different psychotropic class medication exposure in the year after cluster B personality disorder (PD) diagnosis.

Characteristics	Psychotropic medication class																			
	Antipsychotics				Antidepressants				Anxiolytics				Mood stabilizers				ADHD medications			
	PR	99% CI		*P* value	PR	99% CI		*P* value	PR	99% CI		*P* value	PR	99% CI		*P* value	PR	99% CI		*P* value
Age																				
14–24	1	-	-	-	1	-	-	-	1	-	-	-	1	-	-	-	1	-	-	-
25–49	0.98	0.95	1.01	0.0592	**1.30**	**1.26**	**1.34**	**<0.0001**	**1.92**	**1.83**	**2.01**	**<0.0001**	**1.34**	**1.26**	**1.44**	**<0.0001**	**0.51**	**0.47**	**0.55**	**<0.0001**
50–64	0.97	0.94	1.01	0.0296	**1.51**	**1.46**	**1.56**	**<0.0001**	**2.65**	**2.52**	**2.79**	**<0.0001**	**1.63**	**1.51**	**1.75**	**<0.0001**	**0.18**	**0.15**	**0.21**	**<0.0001**
65+	**0.93**	**0.90**	**0.97**	**<0.0001**	**1.36**	**1.32**	**1.41**	**<0.0001**	**2.84**	**2.70**	**2.99**	**<0.0001**	**1.24**	**1.14**	**1.34**	**<0.0001**	**0.06**	**0.05**	**0.08**	**<0.0001**
Sex																				
Male	**1.07**	**1.05**	**1.10**	**<0.0001**	**0.80**	**0.78**	**0.81**	**<0.0001**	**0.85**	**0.83**	**0.87**	**<0.0001**	0.97	0.93	1.01	0.0331	**1.09**	**1.01**	**1.17**	**0.0030**
Female	1	-	-	-	1	-	-	-	1	-	-	-	1	-	-	-	1	-	-	-
Material deprivation^*^ (quintile)																				
1 (least deprived)	1	-	-	-	1	-	-	-	1	-	-	-	1	-	-	-	1	-	-	-
2	**1.09**	**1.04**	**1.14**	**<0.0001**	1.01	0.98	1.05	0.3586	0.99	0.95	1.04	0.6813	1.04	0.96	1.13	0.1623	0.95	0.83	1.09	0.3135
3	**1.08**	**1.04**	**1.13**	**<0.0001**	**1.04**	**1.01**	**1.08**	**0.0022**	1.04	1.00	1.09	0.0195	1.06	0.98	1.15	0.0499	0.94	0.83	1.07	0.2187
4	**1.14**	**1.10**	**1.19**	**<0.0001**	**1.05**	**1.01**	**1.09**	**0.0003**	**1.05**	**1.01**	**1.10**	**0.0020**	1.06	0.98	1.14	0.0516	0.97	0.85	1.10	0.5145
5 (most deprived)	**1.13**	**1.09**	**1.18**	**<0.0001**	**1.07**	**1.03**	**1.11**	**<0.0001**	**1.05**	**1.01**	**1.10**	**0.0009**	1.04	0.97	1.12	0.1509	0.95	0.84	1.07	0.2607
Social deprivation^*^ (quintile)																				
1 (least deprived)	1	-	-	-	1	-	-	-	1	-	-	-	1	-	-	-	1	-	-	-
2	1.02	0.97	1.06	0.3530	1.01	0.97	1.04	0.6823	0.99	0.95	1.04	0.6452	0.97	0.90	1.05	0.3703	1.01	0.88	1.16	0.8151
3	1.01	0.97	1.06	0.4164	1.02	0.98	1.05	0.2861	1.02	0.97	1.07	0.2963	0.93	0.86	1.01	0.0186	1.06	0.93	1.21	0.2286
4	1.01	0.96	1.05	0.7076	1.01	0.98	1.05	0.3211	1.02	0.98	1.06	0.2510	**0.90**	**0.83**	**0.97**	**0.0002**	1.06	0.94	1.20	0.2301
5 (most deprived)	1.02	0.98	1.07	0.1559	1.03	1.00	1.07	0.0111	**1.05**	**1.01**	**1.10**	**0.0021**	**0.89**	**0.83**	**0.96**	**<0.0001**	1.07	0.95	1.21	0.1346
Residence area^$^																				
CMA Montreal	1	-	-	-	1	-	-	-	1	-	-	-	1	-	-	-	1	-	-	-
Other CMA	**1.80**	**1.62**	**1.99**	**<0.0001**	**0.81**	**0.69**	**0.96**	**0.0010**	**1.84**	**1.65**	**2.07**	**<0.0001**	**2.80**	**2.33**	**3.36**	**<0.0001**	0.62	0.29	1.33	0.1089
Agglomerations	**1.10**	**1.07**	**1.14**	**<0.0001**	**1.12**	**1.10**	**1.15**	**<0.0001**	**1.37**	**1.33**	**1.41**	**<0.0001**	**1.19**	**1.13**	**1.25**	**<0.0001**	**1.14**	**1.04**	**1.25**	**0.0001**
Small town/rural	**1.09**	**1.05**	**1.12**	**<0.0001**	**1.12**	**1.09**	**1.15**	**<0.0001**	**1.26**	**1.22**	**1.30**	**<0.0001**	**1.11**	**1.05**	**1.18**	**<0.0001**	**1.13**	**1.02**	**1.25**	**0.0014**
Psychiatric comorbidities^&^																				
Schizophrenia	**1.95**	**1.89**	**2.01**	**<0.0001**	**0.76**	**0.73**	**0.79**	**<0.0001**	**1.18**	**1.14**	**1.22**	**<0.0001**	**1.52**	**1.44**	**1.61**	**<0.0001**	**0.57**	**0.48**	**0.67**	**<0.0001**
Other psychotic disorder	**1.45**	**1.41**	**1.49**	**<0.0001**	**0.83**	**0.81**	**0.86**	**<0.0001**	0.98	0.95	1.02	0.2782	**1.17**	**1.11**	**1.24**	**<0.0001**	**0.87**	**0.75**	**1.00**	**0.0097**
Depression	**1.13**	**1.10**	**1.16**	**<0.0001**	**1.64**	**1.61**	**1.68**	**<0.0001**	**1.22**	**1.19**	**1.26**	**<0.0001**	**1.09**	**1.04**	**1.14**	**<0.0001**	0.94	0.88	1.02	0.0566
Bipolar disorder	**1.38**	**1.35**	**1.42**	**<0.0001**	**1.14**	**1.12**	**1.17**	**<0.0001**	**1.17**	**1.14**	**1.21**	**<0.0001**	**2.94**	**2.81**	**3.07**	**<0.0001**	1.08	0.98	1.19	0.0383
Anxiety disorder	**1.09**	**1.07**	**1.12**	**<0.0001**	**1.34**	**1.32**	**1.37**	**<0.0001**	**1.49**	**1.46**	**1.53**	**<0.0001**	1.01	0.97	1.06	0.3952	1.03	0.96	1.10	0.3148
Adaptive disorder	0.98	0.96	1.00	0.0337	**1.05**	**1.03**	**1.07**	**<0.0001**	**1.04**	**1.01**	**1.07**	**0.0002**	**0.91**	**0.87**	**0.96**	**<0.0001**	1.04	0.96	1.13	0.2351
Alcohol abuse disorder	**1.12**	**1.08**	**1.15**	**<0.0001**	1.02	0.99	1.05	0.0504	**1.23**	**1.19**	**1.27**	**<0.0001**	0.96	0.91	1.01	0.0497	**0.86**	**0.76**	**0.97**	**0.0011**
Drug abuse disorder	**1.18**	**1.15**	**1.22**	**<0.0001**	**1.15**	**1.12**	**1.17**	**<0.0001**	**1.29**	**1.25**	**1.33**	**<0.0001**	1.04	0.99	1.10	0.0436	1.05	0.95	1.15	0.2026
ADHD	**1.21**	**1.15**	**1.28**	**<0.0001**	1.00	0.95	1.05	0.9342	**0.77**	**0.71**	**0.84**	**<0.0001**	0.99	0.88	1.12	0.8930	**5.92**	**5.44**	**6.44**	**<0.0001**
Eating disorder	1.09	0.92	1.30	0.1837	1.12	0.97	1.29	0.0468	1.12	0.91	1.37	0.1591	1.11	0.77	1.60	0.4630	0.75	0.43	1.29	0.1674

B-PD, cluster B personality disorder; CI, Confidence Interval; CMA, census metropolitan area; PR, Prevalence Ratio. In bold: results statistically significant at *p* < 0.01; *Pampalon index at cluster B-PD diagnosis; ^$^Montreal CMA: Montreal and Laval; Other CMA: Quebec, Sherbrooke, Trois-Rivières, Saguenay and Gatineau; ^&^Psychiatric comorbidities in the five years before B-PD diagnosis.

Briefly, we found that age was associated with all the psychotropic classes except antipsychotics, for which only 65 years and older were at lower risk than those 14–24 years old. For anxiolytics, exposure was more likely to occur for older individuals, while for ADHD medications, it happened more frequently for younger individuals. Individuals aged 50 to 64 were more likely to be exposed to antidepressants and mood stabilizers than the youngest. Still, the risk of exposure was generally constantly higher across older age groups.

Being a man was associated with a slightly higher risk of exposure to antipsychotics and ADHD medications (PR: 1.07, 99% CI: 1.05–1.10 and PR: 1.09, 1.01–1.17, respectively) and with a lower risk of exposure to antidepressants and anxiolytics (PR: 0.80, 0.78–0.81 and PR: 0.85, 0.83–0.87, respectively).

Social deprivation was generally not associated with exposure to psychotropics, with few exceptions for anxiolytics and mood stabilizers for the most deprived quintiles. On the contrary, there was a slight increase in the risk of exposure to antidepressants and antipsychotics, and partly to anxiolytics, for increasing material deprivation.

For antipsychotics, the main comorbid conditions associated with a higher risk of exposure were schizophrenia (PR: 1.95), other psychotic disorders (PR: 1.45), bipolar disorders (PR: 1.38), ADHD (PR: 1.21), drug abuse disorders (PR: 1.18), and depression (PR: 1.13). Depression, anxiety disorders, drug abuse disorders, bipolar disorders, and adaptive disorders were associated with an increased risk of exposure to antidepressants, with PRs from 1.64 to 1.05. In contrast, schizophrenia and other psychotic disorders were associated with a lower risk of exposure to this class of medications (PR: 0.76 and PR: 0.83, respectively).

## 4 Discussion

To the best of our knowledge, this is the first study comparing medication use before and after cluster B PD diagnosis in a large cohort of newly diagnosed individuals, with trends and patterns analyzed over a period of 16 years. One of the most striking findings in our study was the extremely high proportion of individuals with cluster B PD receiving psychotropic medications both before and after the first recorded diagnosis, with almost four out of five patients being exposed to at least one psychotropic medication in the year after the diagnosis and a mean 2.8 different psychotropics used. Other studies reported similar high proportions (20–23, 25–31). Moreover, the formal diagnosis of cluster B PD did not seem to reduce the use of psychotropic medications but rather increased the use of some psychotropic medication classes. For those diagnosed with cluster B PD in 2018–2019, the proportion of users remained relatively stable or increased only slightly between 1 year before and after the formal diagnosis for anxiolytics, mood stabilizers, and ADHD medications, with a small peak around the date of the diagnosis for anxiolytics. For antidepressants and antipsychotics, on the contrary, this proportion increased just around the date of the diagnosis and remained higher the entire year afterwards. The peak in the proportion of users around the date of the diagnosis may indicate that the diagnosis of cluster B PD was made after some crisis that required psychotropic medications to control for manifested symptoms, which was a prelude to formal cluster B PD diagnosis.

Nevertheless, while the proportion of users decreased after this crisis period for anxiolytics, it remained stable for antidepressants and, especially, for antipsychotics. Other studies reported extensive antipsychotic utilization, especially atypical, in individuals with PDs (22, 26, 28, 30, 31, 36). While the efficacy of antidepressants seems limited in cluster B PD patients (9), that of antipsychotics, like quetiapine, has been suggested for managing anger, impulsivity and aggressivity (9). This, along with using quetiapine for insomnia, could explain the higher proportion of antipsychotic users after cluster B PD diagnosis (37). The increase in antidepressants and antipsychotic users concurrently with the diagnosis of cluster B PD was especially manifest in younger individuals. Still, it was not neglectable also in older individuals who already had high use. Older individuals were also exposed to anxiolytics in a higher proportion than other age groups, but this was not (or only slightly) affected by cluster B PD diagnosis. It is thus possible that anxiolytics have been used for a long time in older individuals as their use was more prevalent in the past years (38).

Different patterns of psychotropic medication classes use occurred in the last decades. Antidepressants remained the most used medication class, even if their use remained relatively stable over the study period, with only slight differences in the proportion of users depending on the year of diagnosis. Depression and anxiety, the main indications for antidepressant medications (39), were the most frequent comorbid conditions in our sample, with a prevalence of 50.9% for depression and 49.7% for anxiety, often concurrently in the same individual. Nevertheless, no recent clinical trial has been conducted to assess the efficacy of antidepressants in treating cluster B PD (9). Consequently, beyond treating an anxious-depressive comorbid condition, the use of antidepressants for cluster B PDs may have been less frequent or relatively stable.

Antipsychotics showed increased use from 2002 to 2008, remained stable until 2014, and decreased afterwards. In our population, the prevalence of psychotic disorders in the five years before the cluster B PD diagnosis was estimated at 37.5%. For those who were prescribed at least one antipsychotic within a year following their PD diagnosis, the prevalence of schizophrenia, other psychosis, or bipolar disorders was 46.20%. This suggests that more than half of the newly diagnosed PD patients lacked any recorded diagnosis for which an antipsychotic is indicated. Thus, there is a discrepancy between antipsychotic use and recorded diagnoses, which may suggest overuse of medications. However, some differences may result from the lack of diagnoses in the database. Moreover, cluster B PD diagnosis seems to act as a catalyst for antipsychotic prescriptions because of the gap between the proportion of users of these medications before and after the diagnosis. Nonetheless, this gap narrowed slightly in recent years. This pattern could be due to better compliance with clinical guidelines recommending psychotherapy, rather than pharmacotherapy, for cluster B PD treatment and the lack of evidence on antipsychotic treatment efficacy in these individuals (11, 12, 40).

On the other hand, the use of anxiolytics and mood stabilizers has diminished markedly over the study period. The decrease in the utilization of these classes may be related to the increased focus on providing a comprehensive continuum of care for people with PDs. This approach includes psychotherapy, rehabilitation, and all other necessary services across various lines of care (41). Nonetheless, no significant change in the care of patients with mental disorders has been implemented in recent years in Quebec. Moreover, in Quebec, public institutions do not offer coverage for psychotherapy services, leaving residents without personal or work insurance to pay out of pocket for these services. As a result, some individuals cannot access the resources they require, with only a minority of individuals with a mental health problem reporting having sought and received support services (42–44). This significant change could thus be driven by recent clinical guidelines not recommending anxiolytics, such as benzodiazepines, for current mental disorders, with a preference for newer antidepressants (e.g., selective serotonin reuptake inhibitors [SSRIs]) and the consequent change in clinical practice. Indeed, benzodiazepines, the most used anxiolytics, have been associated with injurious falls, fractures, delirium, and long-term cognitive decline, especially in older adults due to their sedative, cognitive-impairing, and motor-impairing effects (45, 46). Benzodiazepines have also been associated in Scandinavian countries’ health administrative databases with the worst health outcomes for schizophrenia, personality disorders, and some substance use disorder (47–50) The decline in the use of anxiolytics has also been reported in older individuals with schizophrenia and in the general aged population in two studies analyzing trends in more than a decade in Quebec (38, 51). Nevertheless, the decline in anxiolytic use we noticed was not compensated for by an equivalent increase in antidepressant use. This suggests that anxiolytics were used more in later years to treat symptomatic aspects of cluster B PDs rather than comorbid anxiety. As for mood stabilizers, their efficacy has not been proven, and their use should be limited to patients with comorbid bipolar disorders (11, 12, 40).

Medications for ADHD showed a constant increase in prescriptions over the study period, from about 1% in 2002–2003 to almost 12% in 2018–2019. This increase aligns with the rise in ADHD diagnosis over the last years in Quebec among children and adults, independently of the diagnosis of cluster B PDs (52, 53). As expected, their use was exceptionally high in younger individuals in recent years, with a proportion of users reaching more than 20% in 2018–2019 (results not shown). Beyond a better recognition of this disorder during the study period, the increased use of ADHD medications could also be due to the favorable risk/benefit profile these medications have shown in clinical practice in reducing ADHD symptoms like hyperactivity and impulsivity (54), with more recent studies also supporting their effectiveness in lowering unintentional injuries (55) and mortality (56), also in PD patients (49).

Trends and patterns in of psychotropic classes combinations first showed that they were more common than single classes. Even if the patterns of combinations changed over time, the proportion of cluster B PD individuals exposed to more than one psychotropic medication only slightly decreased over time (71% in 2002–2003 to 67% in 2018–2019 among users of at least one psychotropic medication). Because of the lack of solid evidence of the effectiveness of psychotropics in cluster B PD individuals, the use of combinations of psychotropic medications raises concerns as they may complexify the therapy of these patients and increase the risk for non-adherence, adverse effects and drug–drug interactions. These concerns are exceptionally elevated in older individuals as they are already at higher risk for multimorbidity and polypharmacy (57–60), which can lead to possible interactions with psychotropic medications, adverse effects and mortality.

Many factors have been associated with exposure to different psychotropic medication classes. Younger age was associated with the use of ADHD medications, which is in line with trends and patterns of diagnosis and treatment of ADHD described in recent studies (61). Age was also associated with exposure to other medication classes, such as antidepressants, antipsychotics, anxiolytics, and mood stabilizers, but in these cases, older individuals were more likely to use them. Generally, those with the higher risk were individuals in the 50–64 years groups, but it was possible to identify a trend for anxiolytics, with an increased risk with increasing age. In a recent study on patients with cluster B PDs, the authors found that older individuals were more likely to use many medications (more elevated than those <65 years) and significantly higher than their peers without cluster B PD diagnosis (62). A cluster B PD diagnosis seems to be a risk factor for receiving many medications, more so than for patients with other psychiatric diagnoses, such as affective disorders (62).

Rather than social deprivation, material deprivation was associated with a higher likelihood of exposure to antipsychotics, antidepressants, and anxiolytics. Our cohort of publicly insured patients was already composed of generally more materially deprived individuals than the general population because, in Quebec, public insurance is offered to those without private employ-related insurance (e.g., unemployed), along with those retired. Nevertheless, the association we found between material deprivation and exposure to psychotropic medications aligns with recent reports for antipsychotic medications in a large retrospective study in the United Kingdom (36). In that study, less deprived individuals with cluster B PDs were less likely to receive antipsychotics than more deprived ones, with adjusted risk ratios between 0.56 and 0.66 (36). More affluent patients may have access to private insurance for psychotherapy since private psychologists are not covered by the public health plan like in the United Kingdom (53).

This study has many strengths. First, it was the first to estimate psychotropic medication use in a large cohort of cluster B PDs individuals throughout the Quebec province. Additionally, the assessment of medication usage prior to and following the initial diagnosis of cluster B personality disorder had not previously been conducted. We thus could find that a not negligible proportion of cluster B PD individuals seemed exposed to psychotropic medications in the period near cluster B PD diagnosis and that this proportion increased after the formal diagnosis. Furthermore, we had access to data spanning nearly two decades, allowing us to analyze trends and patterns in psychotropic medication use. Finally, to identify all the individuals with a registered cluster B personality disorder diagnosis in the QICDSS databases, we used a case definition previously developed through a consensus-leading procedure by a team of experts psychiatrists and psychologists treating PD and using ICD-9 and ICD-10 codes related to DSM symptoms and relevant for clinical practice in Quebec (3).

Nonetheless, the results should be interpreted considering some limitations. Since we used administrative data and claims as a proxy of medication use, we could have overestimated the proportion of people under psychotropic medications. However, our results are consistent with past studies in different countries and settings, suggesting high medication use in cluster B PD individuals. Even in case of an overestimation of medication use, the trends over the 16 years of the study would not have been affected, and the temporal changes observed over time should be accurate. Moreover, the identification of patients with a diagnosis of cluster B PD in the database was based on a case definition using ICD-9 and ICD-10 codes. Since there are no specific ICD codes for cluster B PDs, the codes were chosen through consensus among clinicians. However, the case definition was not formally validated but reflected the consensus reached by a team of experts in the field who treat patients with PDs in Quebec hospitals and clinics. This means that some individuals with cluster A or C may have been misclassified as cluster B PDs due to our definition. Notably, the medico-administrative databases of Quebec, such as QICDSS, are recognized for their high specificity in identifying chronic conditions (33, 63). A form of external validation comes from the important excess mortality we observed in a previous study associated with our definition (3), which is in line with the findings in other countries with comparable linked health administrative databases (64). Moreover, it is worth noting that certain PD patients’ first diagnosis may not have been recorded in the QICDSS because they received it before 1996, the year of developing the QICDSS database, or because they were previously diagnosed in the private sector. To counteract this potential bias, we used a 5-year period to search for diagnosis before the first cohort entry in 2002. Finally, we excluded individuals with private insurance in the two years around cluster B PD diagnosis since the QICDSS database registers only medication use in those under the public drug plan. We cannot thus exclude that medication use would be different in those with private insurance as they are generally less materially deprived and could therefore have better access to psychotherapy in the private sector.

The results of this study indicate that individuals diagnosed with cluster B personality disorders are being prescribed psychotropic medication to a significant extent, especially in the months around the date of diagnosis, which may not be consistent with current best practice recommendations and guidelines. The high use of psychotropic medications may reflect attempts to manage comorbid suicidality and substance use disorders, frequent in cluster B PD patients (3, 5, 6, 65). According to a recent Scandinavian health administrative database study, the long-term benefit of antipsychotics, antidepressants, or benzodiazepines in PD patients was not supported, while the exposure to ADHD medications, clozapine, and bupropion showed a positive effect on these patients (49). A similar and challenging finding was found for patients with methamphetamine use disorder (48), suggesting that the impulsivity dimension present in both PD and substance use disorder (SUD) was regulated by ADHD medication. Medication regulating the craving\sensation seeking dimension of PD and SUD shall also be similarly explored (66, 67). These findings suggest potential challenges to existing guidelines and reflection on the behavioral dimensions of PD and SUD (67), brought by long-term real-life observation made possible by registers of health administrative databases.

We could highlight different trends and patterns in specific psychotropic medication classes over the study period, suggesting essential changes in clinical practice related to certain changes in the management of patients with PDs and more general changes due to better knowledge of psychotropics’ efficacy and safety profiles. Similarly, combinations of psychotropics have been used frequently, with differences in clinical practice during the last sixteen years. In light of these findings, it is vital to conduct further research on the impact of the use of psychotropic medications on health-related outcomes in different healthcare settings, to explore the combination of medications as well as craving\sensation seeking pharmacotherapies.

## Data availability statement

The datasets presented in this article are not readily available because no permission is granted to use the Quebec Integrated Chronic Diseases Surveillance System (QICDSS) data. This means we will not be able to share data. Requests to access the datasets should be directed to Louis.Rochette@inspq.qc.ca.

## Ethics statement

This study is part of the continuous chronic disease surveillance mandate granted to the National Public Health Institute of Quebec (Institut National de Santé Publique du Québec - INSPQ) by the provincial Ministry of Health and Social Services. Ethical approval and written informed consent to participate in this study were not required from the participants or the participants’ legal guardians/next of kin in accordance with the national legislation and the institutional requirements.

## Author contributions

AL, CL, and LC conceived the study and developed the protocol. CS, ER, and VM substantially contributed to the development of the methodology. EL and LR performed the statistical analyses. CL and CM wrote the first draft of the manuscript. AL, CS, EL, ER, EV, LC, LR, MK, RB, PD, PV, SR, and VM revised it critically and substantially contributed to the submitted manuscript. All the authors approved the final version of the manuscript.
