# Supplementary material for: Psychotropic medication use pre and post-diagnosis of cluster B personality disorder: a Quebec’s health services register cohort
Source: Front Psychiatry. 2023 Nov 23;14:1243511. doi: 10.3389/fpsyt.2023.1243511 (PMC10702219; doi:10.3389/fpsyt.2023.1243511)
Supplement: Supplementary file 1 [file Table_1.DOCX]

Supplementary Material

Psychotropic Medication Use Before and After a Diagnosis of Cluster B Personality Disorder: a Quebec’s health services register cohort from 2002 to 2018

**Carlotta Lunghi*, Lionel Cailhol, Victoria Massamba, Elhadji A. Laouan Sidi, Caroline Sirois, Elham Rahme, Louis Rochette, Suzane Renaud, Evens Villeneuve, Marion Koch, Robert Biskin, Cathy Martineau, Philippe Vincent, Pierre David, Alain Lesage**

*** Correspondence:** Carlotta Lunghi: [carlotta_lunghi@uqar.ca](mailto:carlotta_lunghi@uqar.ca); [carlotta.lunghi@unibo.it](mailto:carlotta.lunghi@unibo.it)

## Supplementary Figures

**Supplementary Figure 1. Mean number of psychotropic medications used in the year after cluster B personality disorder (PD) diagnosis, according to psychotropic class and the year of PD diagnosis.**

**Supplementary Figure 2. Proportion of individuals exposed to typical and atypical antipsychotics in the year before and after a cluster B personality disorder (PD) diagnosis** Solid lines represent the proportion of patients exposed to medications in the year before cluster B PD diagnosis, and dashed lines represent the proportion of patients exposed to medications in the year after the diagnosis.

## Supplementary Tables

**Supplementary Table 1 Frequency of the combination of psychotropic medications claimed in the year after cluster B personality disorder (PD) diagnosis (Fiscal years 2002 vs 2018)**

| **Combinations** | **Before B-PD diagnosis (2002)** | | **After B-PD diagnosis (2002)** | | **Before B-PD diagnosis (2018)** | | **After B-PD diagnosis (2018)** | |
| --- | --- | --- | --- | --- | --- | --- | --- | --- |
|  | **N^#^** | **%** | **N^#^** | **%** | **N^#^** | **%** | **N^#^** | **%** |
| DEP - ANX | 1100 | 27.10 | 960 | 22.20 | 340 | 11.90 | 290 | 9.58 |
| DEP | 660 | 16.25 | 600 | 13.96 | 580 | 20.21 | 535 | 17.52 |
| ANX | 440 | 10.92 | 380 | 8.80 | 125 | 4.28 | 95 | 3.19 |
| PSY - DEP - ANX | 440 | 10.83 | 590 | 13.68 | 340 | 11.83 | 375 | 12.38 |
| PSY - DEP - ANX - MOOD | 245 | 5.99 | 330 | 7.73 | 95 | 3.30 | 125 | 3.99 |
| DEP - ANX - MOOD | 215 | 5.38 | 205 | 4.67 | 40 | 1.39 | 35 | 1.19 |
| PSY - ANX | 195 | 4.91 | 220 | 5.16 | 85 | 3.10 | 75 | 2.37 |
| PSY | 150 | 3.72 | 200 | 4.55 | 180 | 6.26 | 250 | 8.10 |
| PSY - ANX - MOOD | 130 | 3.21 | 185 | 4.30 | 40 | 1.39 | 45 | 1.58 |
| PSY - DEP | 125 | 3.06 | 200 | 4.58 | 395 | 13.67 | 485 | 16.11 |
| DEP - MOOD | 65 | 1.60 | 75 | 1.72 | 45 | 1.57 | 55 | 1.88 |
| PSY - MOOD | 60 | 1.53 | 105 | 2.37 | 35 | 1.22 | 40 | 1.35 |
| PSY - DEP - MOOD | 55 | 1.36 | 80 | 1.76 | 50 | 1.81 | 85 | 2.83 |
| ANX - MOOD | 55 | 1.36 | 60 | 1.46 | 30 | 1.11 | 25 | 0.86 |
| MOOD | 50 | 1.33 | 50 | 1.21 | 60 | 2.05 | 55 | 1.91 |
| ADHD | 20 | 0.42 | 10 | 0.30 | 75 | 2.57 | 65 | 2.27 |
| DEP - ADHD | 10 | 0.17 | 15 | 0.33 | 80 | 2.82 | 85 | 2.80 |
| DEP - ANX - ADHD | 5 | 0.17 | 10 | 0.23 | 35 | 1.11 | 25 | 0.82 |
| PSY - DEP - ANX - MOOD - ADHD | 10 | 0.15 | 10 | 0.23 | 10 | 0.35 | 20 | 0.69 |
| PSY - DEP - ANX - ADHD | 5 | 0.12 | 10 | 0.23 | 50 | 1.60 | 45 | 1.52 |
| DEP - ANX - MOOD - ADHD | 5 | 0.12 | 5 | 0.12 | 10 | 0.28 | 5 | 0.26 |
| PSY - DEP - ADHD | 0 | 0.07 | 0 | 0.09 | 95 | 3.23 | 105 | 3.46 |
| PSY - ADHD | 5 | 0.05 | 0 | 0.09 | 40 | 1.36 | 45 | 1.58 |
| PSY - DEP - MOOD - ADHD | 0 | 0.05 | 0 | 0.02 | 5 | 0.21 | 15 | 0.49 |
| PSY - ANX - MOOD - ADHD | 5 | 0.02 | 0 | 0.09 | 5 | 0.10 | 0 | 0.13 |
| MOOD - ADHD | 5 | 0.02 | 5 | 0.02 | 10 | 0.35 | 5 | 0.30 |
| PSY - ANX - ADHD | 5 | 0.02 | 5 | 0.02 | 10 | 0.38 | 10 | 0.30 |
| ANX - ADHD | 0 | 0.02 | 0 | 0.02 | 5 | 0.17 | 0 | 0.07 |
| PSY - MOOD - ADHD | 0 | 0.02 | 0 | 0.02 | 0 | 0.10 | 0 | 0.13 |
| DEP - MOOD - ADHD | 0 | 0.00 | 5 | 0.02 | 10 | 0.21 | 10 | 0.26 |
| ANX - MOOD - ADHD | 0 | 0.00 | 0 | 0.00 | 5 | 0.07 | 5 | 0.07 |

ADHD: medications for ADHD; ANX: anxiolytics; B-PD: cluster B personality disorder; DEP: antidepressants; MOOD: medications for mood disorders; PSY: antipsychotics. ^#^Randomly rounded to 0 or 5 to maintain confidentiality.
