# Supplementary figures and images for: Psychotropic medication use pre and post-diagnosis of cluster B personality disorder: a Quebec’s health services register cohort
Source: Front Psychiatry. 2023 Nov 23;14:1243511. doi: 10.3389/fpsyt.2023.1243511 (PMC10702219; doi:10.3389/fpsyt.2023.1243511)

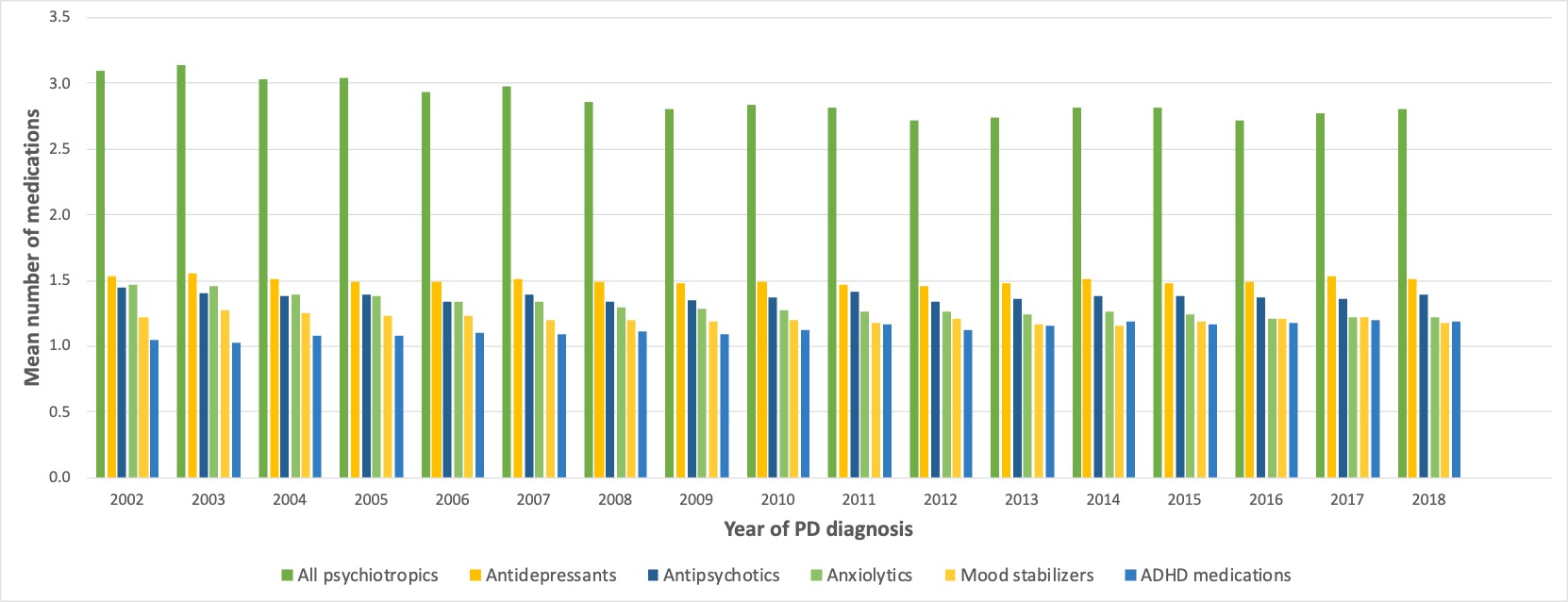

Supplement: Supplementary file 2 [file Image_1.JPEG]

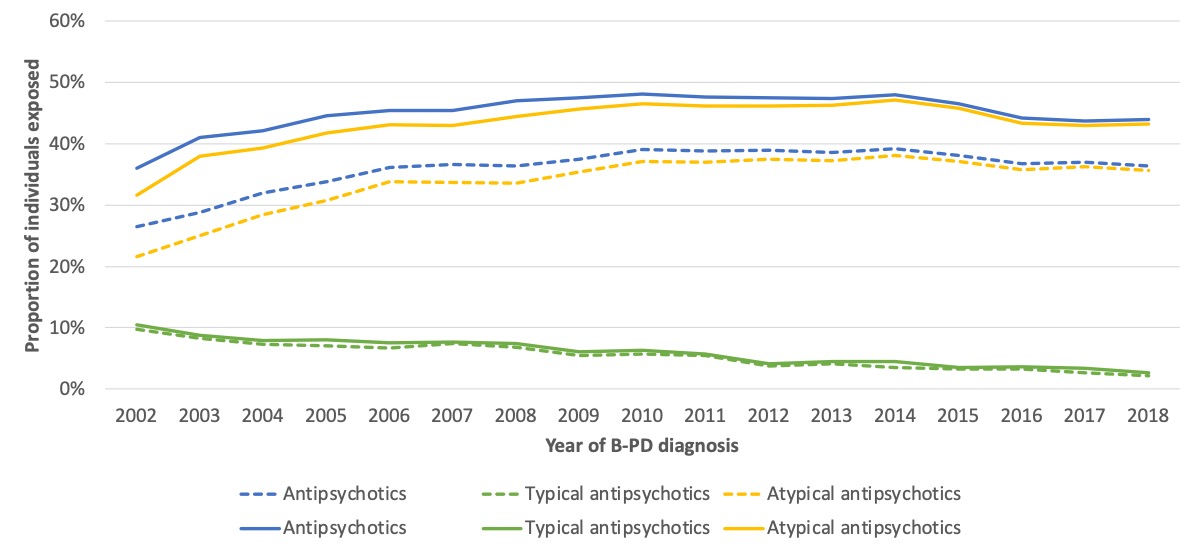

Supplement: Supplementary file 3 [file Image_2.JPEG]
